# The Pathological Features of Common Hereditary Mitochondrial Dynamics Neuropathy

**DOI:** 10.3389/fnins.2021.705277

**Published:** 2021-07-22

**Authors:** Rui Wu, He Lv, Hui Wang, Zhaoxia Wang, Yun Yuan

**Affiliations:** ^1^Department of Neurology, Peking University First Hospital, Beijing, China; ^2^Department of Neurology, Shandong Provincial Hospital, Shandong University, Jinan, China

**Keywords:** mitochondrial dynamics, Charcot–Marie–Tooth Disease, MFN2, GDAP1, sural biopsy

## Abstract

**Objectives:**

Mitofusin 2 and ganglioside-induced differentiation-associated protein 1 are two main mitochondrial dynamics-related proteins. Dysfunction of these two proteins leads to different subtypes of Charcot–Marie–Tooth disease type 2A (CMT2A) and CMT2K. This study aims to report the pathological difference between CMT2A and CMT2K in a large cohort.

**Methods:**

Thirty patients with molecularly confirmed CMT2A and nine with CMT2K were identified by next-generation sequencing. Sural nerve biopsies were performed in 29 patients.

**Results:**

The patients with both diseases showed length-dependent neuropathy with distal weakness, sensory loss, and no deep tendon reflex. Optic neuropathy appeared in 3/30 (10%) patients with CMT2A. Tendon contracture appeared in 4/9 (50.0%) patients with CMT2K. Sural biopsy revealed the loss of both myelinated and unmyelinated nerve fibers. Closely packed, irregularly oriented neurofilaments were observed in axons of unmyelinated nerve fibers in both diseases. Another important finding was the ubiquitous presence of smaller, rounded, and fragmented mitochondria in CMT2A and elongated mitochondria in CMT2K in the myelinated and unmyelinated axons.

**Conclusion:**

This study confirmed large diversity in phenotypes between CMT2A and CMT2K. Mitochondrial dynamics-related variations can induce different mitochondrial morphological changes and neurofilament accumulation in axons.

## Introduction

Charcot–Marie–Tooth disease (CMT), also known as hereditary motor and sensory neuropathy, is the most common type of hereditary peripheral neuropathy with an incidence of about 1/2,500 (Lerat et al., 2019; Tao et al., 2019). Variations related to mitochondrial dynamics, including mitochondrial fusion, fission, transport, and mitophagy, are the leading causes of axonal CMT2 (Pareyson et al., 2015; Minaidou et al., 2019; Sancho et al., 2019). During fusion, normal mitochondria can compensate for the impaired function of mutated ones carrying defective mitochondrial DNA (mtDNA) (especially mtDNA deletion) by supplying normal mtDNA, resulting in functional mitochondria, and then generating normal mitochondria by fission (Georgiou et al., 2017). Therefore, fusion and fission have a certain dilution effect on the mitochondrial genome variations and play an important role in maintaining the integrity of the respiratory chain and cell energy metabolism (El-Hattab et al., 2017). This process disorder can lead to the accumulation of defective mtDNA in the neuron. On the other hand, it can also result in mitochondrial shape change and further block its axonal transport. The impaired transport and uneven distribution of mitochondria result in energy insufficiency especially in the terminal of the long axons and causes axonal damage (Misko et al., 2012; Pareyson et al., 2013).

Mitofusin 2 (*MFN2*) and ganglioside-induced differentiation-associated protein 1 (*GDAP1*) are mitochondrial dynamics-related genes that participate mainly in mitochondrial fusion and fission, respectively. By reviewing the previous cohorts in Germany (Rudnik-Schoneborn et al., 2016), France (Bombelli et al., 2014), Korea (Choi et al., 2015), and Japan (Yoshimura et al., 2017), patients with variations of the two genes are usually characterized by length-dependent peripheral neuropathy. Some patients additionally presented with hearing loss, scoliosis, vocal cord paralysis, diaphragmatic paralysis, cognitive impairment, and pyramidal sign (Martin et al., 2015; Sivera et al., 2017; Iapadre et al., 2018). Nerve conduction study showed low amplitude of compound muscle action potential (CMAP) and sensory nerve action potential (SNAP), with motor nerve conduction velocity (MNCV) relatively preserved (Marttila et al., 2017; Bernard-Marissal et al., 2019). Sural biopsy revealed axonal loss with mitochondrial morphological changes with neurofilament aggregate in axons in CMT2A and CMT2K (Verhoeven et al., 2006; Lv et al., 2015; Fu et al., 2017). The dysfunctional mitochondrial pathways in hereditary neuropathy provide feasible molecular targets for assessing the relationship between mitochondrial dysfunction and other pathological changes in neuropathy. Based on a large Chinese cohort, we study the clinical and pathological characteristics of mitochondrial dynamics-related CMT.

## Materials and Methods

A total of 30 CMT2A and nine CMT2K patients were diagnosed by next-generation sequencing and traditional DNA sequencing from January 2007 to May 2020 at the Department of Neurology, Peking University First Hospital. The detailed clinical data (including age at onset, disease duration, degree and distribution of weakness, muscle atrophy, sensory loss, tremor, tendon reflexes, foot deformities, and family history) were retrospectively collected and analyzed after giving informed consent. Nerve conduction studies were performed in 31 patients (23 with *MFN2* variants and eight with *GDAP1* variants). The CMT Neuropathy Score (CMTNS) was calculated according to the clinical and electrophysiological features to evaluate clinical severity, which was defined as mildly impaired (CMTNS2 ≤ 10), moderately impaired (CMTNS2 11–20), and severely impaired (CMTNS2 21–36) (Murphy et al., 2011).

Sural nerve biopsies were done in 29 patients (23 with MFN2 variations and six with GDAP1 variations), and the nerve specimens were processed using standard methods as follows: a larger piece of the specimen was fixed in 4% formalin, embedded in paraffin, and stained with hematoxylin–eosin, Congo Red, and Luxol Fast Blue. Immunohistochemistry staining includes myelin basic protein (MBP) and neurofilament (NF). The residual piece was fixed in 3% glutaraldehyde and postfixed in 1% osmium tetroxide. Semithin sections were stained with toluidine blue for observation under the light microscope. Ultrathin sections for electron microscopy were contrasted with uranyl acetate and lead citrate. The nerve fiber density and diameter were calculated using NIS-Elements BR 3.2 program.

Data statistics were analyzed using SPSS 23.0 software. Numerical data were expressed as means and medians, and categorical data were expressed as percentiles. The *t*-test was used to compare the age at onset, disease duration, MNCV, amplitude of CMAPs and SNAPs, and CMTNS scores between the two groups. Fisher’s exact test was processed to compare the differences in the rate of clinical and pathological features. All comparisons were statistically significant with *p* < 0.05.

## Results

### Clinical Features of Mitochondrial Dynamics-Related Charcot–Marie–Tooth Disease

In a total of 160 patients clinically suspected of CMTs, we found 30 patients with the MFN2 prevalence rate of 18.8% (75% of CMT2s) and GDAP1 prevalence rate of 5.6% (21.4% of CMT2s). Thirty cases carrying *MFN2* variants were classified as CMT2A, and nine cases carrying *GDAP1* variants were classified as CMT2K according to the clinical manifestations and electrophysiological data. The clinical features of CMT2A and CMT2K are summarized in Table 1. All patients showed length-dependent motor and sensory neuropathy. CMT2A has significantly higher rates of *pes cavus* (*p* < 0.01) and distal muscle atrophy in upper extremities (*p* = 0.02). CMT2K has significantly higher rates of Achilles tendon contracture (*p* < 0.01) than CMT2A. Besides, optic neuropathy with visual impairment only occurred in CMT2A, while tendon contracture and vocal cord paralysis are only seen in CMT2K. There are no significant differences between the two groups of patients in terms of gender, age at onset, disease duration, rates of muscle weakness, sensory disorders, and decreased reflexes. The CMTNS2 scores are also not significantly different between the two groups as well as the percentage of severely impaired cases.

**TABLE 1:** Comparison of clinical features of the patients with CMT2A and CMT2K.

	***MFN2***	***GDAP1***	***p*-value**
Male (*n*, %)	21 (72.4)	4 (44.4)	0.12
Age at onset (years)	5.3 ± 6.4 (1–26)	4.6 ± 2.6 (1–10)	0.10
Disease duration (years)	9.7 ± 9.6 (0.5–37)	14.9 ± 12.8 (4–36)	0.16
Distal weakness (*n*, %)			
Lower limbs	27 (100.0)	8 (100.0)	–
Upper limbs	15 (55.6)	5 (62.5)	0.73
Distal atrophy (*n*, %)			
Lower limbs	27 (100.0)	8 (100.0)	–
Upper limbs*	16 (59.3)	1 (12.5)	0.02
*Pes cavus* (*n*, %)*	25 (92.6)	2 (25.0)	<0.01
Tendon contracture*	0 (0.0)	4 (50.0)	<0.01
Decreased pin perception (*n*, %)	21 (77.8)	8 (100.0)	0.14
Decreased vibration (*n*, %)	21 (77.8)	8 (100.0)	0.14
CMTNS2	15.5 ± 6.4	14.7 ± 7.3	0.51
Decreased or absent reflex (*n*, %)	27 (100.0)	8 (100.0)	–
Vision impairment (*n*, %)	3 (11.1)	0 (0.0)	0.32
Positive family history (*n*, %)	7 (25.9)	1 (12.5)	0.43
Hoarseness	0	1 (12.5)	–

*Rates of clinical features between two groups are significantly different. CMT, Charcot–Marie–Tooth disease; MFN2, mitofusin 2; GDAP1, ganglioside-induced differentiation-associated protein 1.

### Nerve Conduction Studies

Electrophysiological data were available for 23 CMT2A and eight CMT2K patients (Table 2). All the recordable NCVs of the motor median nerve were not lower than 38 m/s. The median nerve CMAP non-elicited rate is 23.8% in CMT2A and 12.5% in CMT2K. CMT2A cases also have a higher peroneal nerve CMAP non-recordable rate than CMT2K (73.9 vs. 50%).

**TABLE 2:** Nerve conduction study of the patients with CMT2A and CMT2K.

**Patient**	**Median nerve**				**Peroneal nerve**			
	**MNCV**	**CMAP**	**SNCV**	**SNAP**	**MNCV**	**CMAP**	**SNCV**	**SNAP**
**CMT2A**								
1	40.4	0.7	–	–	–	–	–	–
2	–	–	–	–	–	–	–	–
3	51.4	7.3	–	–	–	–	–	–
4	51.6	0.4	–	–	43.4	0.4	–	–
5	–	–	48.6	2.9	–	–	62.2	5.6
6	38.5	3.1	30.4	20.0	23.3	0.3	–	–
7	ND	ND	ND	ND	–	–	–	–
8	57.7	6.6	ND	ND	18.1	0.5	35.3	1.4
9	43.8	4.3	28.6	3.2	–	–	–	–
10	–	–	–	–	–	–	–	–
11	38.0	5.1	35.0	2.0	–	–	–	–
12	51.0	3.6	–	–	–	–	–	–
13	48.1	1.14	–	–	–	–	–	–
14	ND	ND	ND	ND	–	–	–	–
15	52.1	10.6	53.9	7.6	–	–	48.1	0.1
16	55.4	10.6	56.4	10.1	40.0	0.57	–	–
17	57.2	14.6	42.4	3.0	–	–	–	–
18	51.1	3.1	–	–	–	–	–	–
19	42.0	9.8	49.0	6.0	–	–	–	–
20	–	–	–	–	–	–	–	–
21	–	–	–	–	–	–	–	–
22	53.3	3.3	–	–	–	–	–	–
23	43.9	3.2	32.2	4.9	–	–	–	–
**CMT2K**								
1	48.1	3.8	66.7	16.0	34.5	–	–	–
2	45.2	1.0	–	–	–	–	–	–
3	57.3	2.7	14.4	5.5	25.4	0.02	–	–
4	55.0	1.5	–	–	–	–	–	–
5	–	–	–	–	–	–	–	–
6	53.6	3.9	44.9	1.5	30.2	1.18	39.0	6.5
7	56.6	3.5	–	–	–	–	–	–
8	59.2	6.5	–	–	31.2	0.4	–	–

*CMT, Charcot–Marie–Tooth disease; –, absent evoked response; CMAP, compound muscle action potential (the amplitude of CMAP in mV); SNAP, sensory nerve action potential (the amplitude of SNAP in μV); MNCV, motor nerve conduction velocity (in m/s); SNCV, sensory nerve conduction velocity (in m/s); ND, not measured.*

### Pathological Features

**FIGURE 1:**
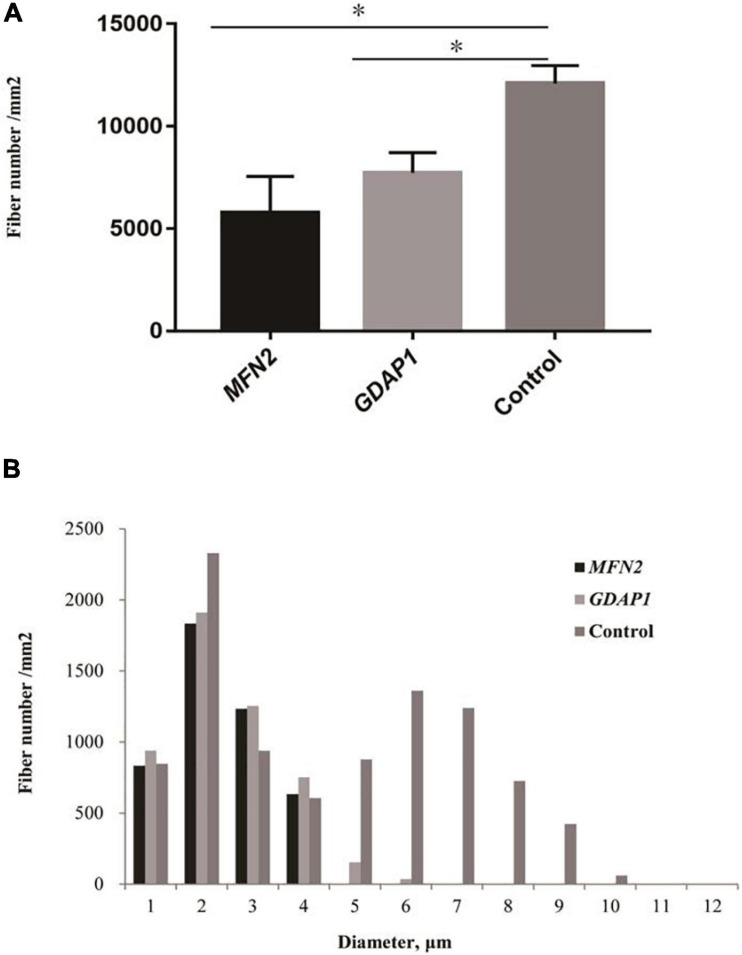
Quantitative analysis of sural biopsy specimens. **(A)** The myelinated fiber density of patients with Charcot–Marie–Tooth disease (CMT)2A and CMT2K compared with age- and sex-matched controls. **(B)** Histogram showing the unimodal distribution pattern of myelinated fibers with loss of large myelinated fibers and low fiber density in patients compared with age- and sex-matched controls. ^∗^*p* < 0.05.

**FIGURE 2:**
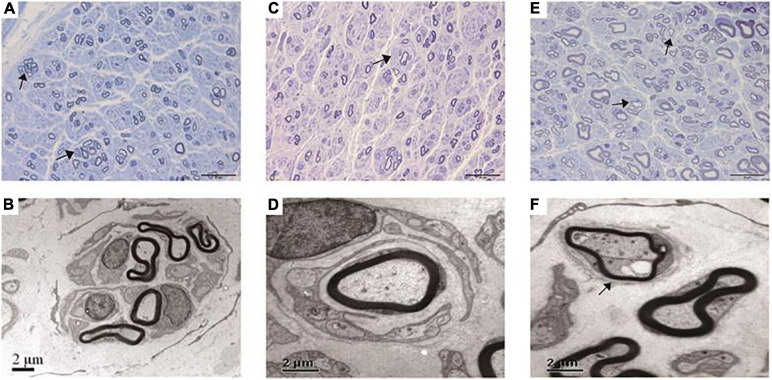
Pathological features under the light microscope and electron microscopy of this cohort. **(A,B)** Regeneration clusters (arrows) in a CMT2A case. **(C,D)** Onion bulbs (arrow) in a CMT2K case. **(E,F)** Thin myelinated fibers (arrows) in a CMT2K case.

The pathological features of the present cases are summarized in Table 3. The myelinated fiber densities of all *MFN2*- and *GDAP1*-associated CMTs are decreased (Figure 1A). Moreover, large myelinated fibers are more severely involved morphometrically compared with age- and sex-matched controls (Figure 1B). Regeneration clusters (Figures 2A,B) can be seen in 21 patients with CMT2A and seven patients with CMT2K. Atypical onion bulbs (Figures 2C,D) and thin myelinated fibers (Figures 2E,F) can be seen in 9/6 patients with CMT2A and 6/3 patients with CMT2K. As shown in Table 3, the rate of atypical onion bulb structure is higher in CMT2K (*p* = 0.03). The residual unmyelinated fibers were loosely distributed without a normal cluster profile in neurofilament immunohistochemistry.

**TABLE 3:** Pathological features of the patients with CMT2A and CMT2K.

	**CMT2A**	**CMT2K**	***p*-value**
MF density/mm^2^	5,785.3 ± 1,767.6 (2,341–7,941)	7,307.0 ± 1,846.9 (6,572–9,026)	0.21
Regeneration clusters of MF (*n*, %)	21 (91.3)	7 (100.0)	0.42
Axonal degeneration (*n*, %)	3 (13.0)	1 (14.3)	0.93
Thin myelinated fibers (*n*, %)	6 (26.1)	3 (42.9)	0.40
Onion bulbs (*n*, %)*	9 (39.1)	6 (85.7)	0.03

*Rates of clinical features between two groups are significantly different. CMT, Charcot–Marie–Tooth disease; MF, myelinated fibers.

**FIGURE 3:**
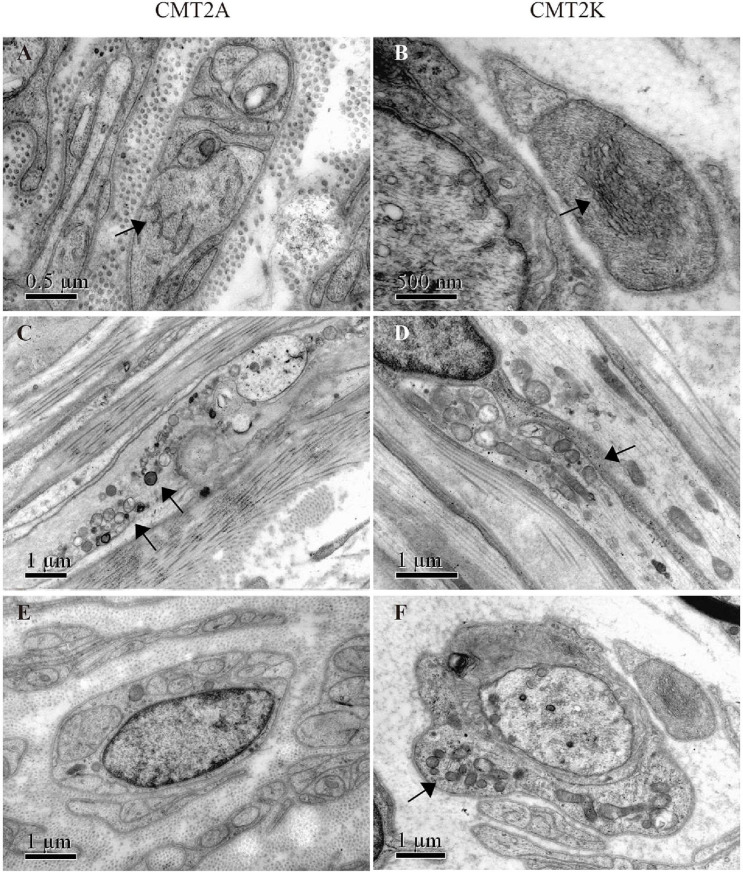
Mitochondrial morphology in mitofusin 2 (MFN2)- and ganglioside-induced differentiation-associated protein 1 (GDAP1)-related CMT. **(A,B)** Closely packed, irregularly oriented neurofilaments in axons of unmyelinated nerve fibers (arrow) in CMT2A and CMT2K. **(C)** Abnormal aggregation of the round, small, and fragmented mitochondria in CMT2A (arrows). **(D)** Mitochondrial elongation and aggregation in CMT2K (arrow). **(E)** Scattered mitochondria in the Schwann cell of CMT2A. **(F)** Aggregation of mitochondria in the Schwann cell of CMT2K (arrow).

Electron microscopy revealed closely packed, irregularly oriented neurofilaments in axons of unmyelinated fibers in both CMT2A and CMT2K axons (Figures 3A,B). Degenerating mitochondria focally accumulate in axons of myelinated and unmyelinated fibers. In CMT2A, the mitochondria showed swelling, condensation into dense bodies, transformation in myeloid, and irregularities of the inner and outer mitochondrial membrane with different diameters. Close contacts between neighboring mitochondria showed no fusion. Most mitochondria showed outer and inner membrane fusion with uneven density (Figure 3C). In addition to small round mitochondria, another group of elongated mitochondria could be seen in axons of CMT2K (Figure 3D). Moreover, mitochondria aggregation is also seen in Schwann cells of CMT2K (Figure 3F), which was not seen in CMT2A (Figure 3E). Unmyelinated fibers were damaged, as evidenced by the appearance of collagen pockets.

### Genetic Findings

The variants of *MFN2* in all 29 CMT2A patients are heterozygous, including 15 missense variants (c.280C > T, c.281G > A, c.292A > G, c.310C > T, c.314C > T, c.493C > T, c.614T > C, c.718T > G, c.730G > T, c.743T > A, c.748C > T, c.775C > T, c.1070A > C, c.1090C > T, c.1100A > C, and c.2218T > C). Fourteen of them are located in the GTPase domain or close to it where variations occur most frequently. Only one (c.2218T > C) is located in the coiled-coil 2 (Cc2) domain, which is the most important structure when MFN1 and MFN2 form a homodimer or heterodimer and is highly conserved among different species. Nine CMT2K patients carried nine missense (c.122G > A, c.358C > T, c.466G > A, c.478A > G, c.563A > G, c.719G > C, c.719G > A, c.767A > G, and c.845G > A) and one frame-shift variant (N210KFS^∗^5) of *GDAP1* gene. There were four with compound heterozygous variants, four with heterozygous variants, and one with homozygous variant. The variants are evenly distributed along GST and α4α5loop domains (Table 4). All the mutations have been reported as pathogenic.

**TABLE 4:** Genotype distribution of *MFN2* and *GDAP1.*

**Patients**	**Gene**	**Nucleotide**	**Amino acid**	**Exon**	**Domain**	**Inheritance**
1	*MFN2*	c.310C > T	R104W	5	GTPase	AD
2	*MFN2*	c.281G > A	R94Q	4	GTPase*	AD
3	*MFN2*	c.314C > T	T105M	5	GTPase	AD
4	*MFN2*	c.1090C > T	R364W	11	GTPase*	AD
5	*MFN2*	c.280C > T	R94W	4	GTPase*	AD
6	*MFN2*	c.1090C > T	R364W	11	GTPase*	AD
7	*MFN2*	c.292A > G	K98E	4	GTPase*	AD
8	*MFN2*	c.730G > T	V244L	8	GTPase	AD
9	*MFN2*	c.614T > C	V205A	7	GTPase	AD
10	*MFN2*	c.1070A > C	K357T	11	GTPase*	AD
11	*MFN2*	c.1100 A > C	Q367P	11	GTPase*	AD
12	*MFN2*	c.1090C > T	R364W	11	GTPase*	AD
13	*MFN2*	c.310C > T	R104W	5	GTPase	AD
14	*MFN2*	c.281G > A	R94Q	4	GTPase*	AD
15	*MFN2*	c.280C > T	R94W	4	GTPase*	AD
16	*MFN2*	c.280C > T	R94W	4	GTPase*	AD
17	*MFN2*	c.280C > T	R94W	4	GTPase*	AD
18	*MFN2*	c.2218T > C	W740R	19	Cc2	AD
19	*MFN2*	c.1090C > T	R364W	11	GTPase*	AD
20	*MFN2*	c.775C > T	R259C	8	GTPase	AD
21	*MFN2*	c.493C > T	H165Y	6	GTPase	AD
22	*MFN2*	c.748C > T	R250W	8	GTPase	AD
23	*MFN2*	c.280C > T,	R94W	4	GTPase*	AD
24	*MFN2*	c.1090C > T	R364W	11	GTPase*	AD
25	*MFN2*	c.718T > G	P240V	8	GTPase	AD
26	*MFN2*	c.718T > G	P240V	8	GTPase	AD
27	*MFN2*	c.743T > A	I248H	8	GTPase	AD
28	*MFN2*	c.314C > T	T105M	5	GTPase	AD
29	*MFN2*	c.280C > T	R94W	4	GTPase*	AD
30	*MFN2*	c.776G > A	R259H	8	GTPase	AD
31	*GDAP1*	c.845G > A c.767A > G	R282H H256R	6 6	GST-C GST-C	AR
32	*GDAP1*	c.767A > G c.466G > A	H256R A156T	6 3	GST-C α4α5 loop	AR
33	*GDAP1*	c.719G > A	C240Y	6	GST-C	AD
34	*GDAP1*	c.358C > T	R120W	3	GST-N	AD
35	*GDAP1*	c.563A > G c.563A > G	H188R H188R	6 6	α4α5 loop α4α5 loop	AR
36	*GDAP1*	c.845G > A c.767A > G	R282H H256R	6 6	GST-C GST-C	AR
37	*GDAP1*	c.122G > A c.533A > G	R41H N178S	2 4	GST-N α4α5 loop	AR
38	*GDAP1*	c.603delT	N210KFS*5	2	GST-N	AD
39	*GDAP1*	c.478A > G	R160V	4	α4α5 loop	AD

MFN2, mitofusin 2; GDAP1, ganglioside-induced differentiation-associated protein 1; GTPase, guanosine triphosphatase domain; GTPase*, close to GTPase domain; Cc2, coiled-coil 2 domain; GST-N, glutathione S-transferase domain in the N-terminal region; GST-C, glutathione S-transferase domain in the C-terminal region; AD, autosomal dominant; AR, autosomal recessive.

## Discussion

Since DiMauro and Schon (2008) proposed the concept of mitochondrial dynamics in 2008, the close relationship between the mechanism and diseases began to attract attention. Mitochondrial fusion–fission are the most common dynamic processes that not only keep the mitochondria in normal size, shape, and number but are more important to maintain the stability of the mitochondrial genome (Lv et al., 2015; Fu et al., 2017). A variety of proteins are involved in this process, and the main ones related to CMT are fusion-related MFN2 and fission-related GDAP1. This study summarizes the clinical and pathological features of the two CMT subtypes in a large cohort.

Cases in this cohort mostly have onset in the first decade, while Japanese CMT2K patients showed a mean onset age of 19.5 years (Yoshimura et al., 2017). All the cases manifest as the length-dependent peripheral neuropathy that weakness, muscle atrophy, and sensory dysfunction are more obvious in the distal limbs (Choi et al., 2015; Rudnik-Schoneborn et al., 2016; Ando et al., 2017). Previously reported concomitant symptoms of CMT2A such as cognitive impairment (Tomaselli et al., 2018), stroke (Tomaselli et al., 2018), spinal cord atrophy (Hikiami et al., 2018), hearing loss (Hikiami et al., 2018), and vocal cord paralysis were not found in our patients (Zuchner et al., 2006). In addition to vocal cord paralysis, accompanying symptoms related to CMT2K such as scoliosis (Kabzinska et al., 2011), pyramidal signs, and diaphragmatic paralysis (Biancheri et al., 2006) are absent in our patients.

There were no cases with MNCVs below 38 m/s in median nerves; hence, there is no demyelinated subtype in the present cohort. This finding was also reported in another Chinese cohort (Biancheri et al., 2006), as well as in studies in other countries (Sevilla et al., 2003; Saporta et al., 2011; Sivera et al., 2013; Yoshimura et al., 2017). We found that the sensory nerve was more involved in CMT2K than the CMT2A. The median nerve CMAP non-recordable rate is higher in CMT2A, which is consistent with the higher incidence of distal upper limb atrophy seen in CMT2A. Such clinical manifestations–electrophysiology correlation was also found in the peroneal nerve in that CMT2A cases that have higher peroneal nerve CMAP non-recordable rates also have higher occurrence rates of *pes cavus*.

Sural biopsies confirmed the moderate to severe loss of large myelinated fibers and appearance of axonal regeneration sprouts, which are similar to those of other studies (De Sandre-Giovannoli et al., 2003; Sevilla et al., 2003; Verhoeven et al., 2006). We noticed that GDAP1 variations affect myelin sheaths more severely than MFN2 variations since CMT2K had a higher percentage of onion bulb structures formed by proliferation of Schwann cells and thin myelinated fibers than CMT2A. The unmyelinated fiber loss appeared mainly in the later stage of CMT2K and CMT2A with similar frequency.

Ultrastructure analysis revealed that the distribution and morphology of mitochondria were correlated with the molecular diagnosis and implied the pathogenic mechanism. The mitochondria accumulated in axons induced by mitochondrial dynamics defects and further resulted in axonal damage (De Sandre-Giovannoli et al., 2003; Sevilla et al., 2003; Verhoeven et al., 2006). In terms of morphology, the mitochondria in CMT2A cases are usually smaller, rounded, and condensed into dense bodies, indicating the block of fusion process in axons (Verhoeven et al., 2006). In comparison, mitochondria in CMT2K’s axons could be elongated, as described in another study that detected that GDAP1 knockdown in neuroblastoma cells causes elongated mitochondria with tubular morphology (Niemann et al., 2005). Combined with the pathological changes we have seen in CMT2K of this cohort, the pathophysiology may be associated with mitochondrial fission disorder and abnormal distribution and movement throughout the cytoskeleton toward the endoplasmic reticulum and subplasmalemmal microdomains (Niemann et al., 2005).

Many neurodegenerative disorders are well known to involve the accumulation of disease-specific proteins. Recent studies provide novel insights into the role of neurofilament accumulation in the more common neurological disorders. Immunostaining for neurofilament confirmed that numerous dilated axon segments were present in MFN2 mutant–consistent with ongoing axonal degeneration in cultured neurons (Niemann et al., 2005). We found neurofilament accumulation in axons of CMT2A and CMT2K patients that suggested that the mitochondrial dynamics dysfunction plays an important role in the development of accumulation of proteins in the neurons. Further study is needed to explain the signal pathway between neurofilament accumulation and MFN2 or GDAP1 variations.

## Conclusion

This study confirmed the large diversity in phenotypes between CMT2A and CMT2K. Mitochondrial dynamics-related gene variation can induce different mitochondrial morphological changes and neurofilament accumulation in axons.

## Data Availability Statement

The original contributions presented in the study are included in the article/Supplementary Material, further inquiries can be directed to the corresponding author.

## Ethics Statement

The studies involving human participants were reviewed and approved by Peking University First Hospital. Written informed consent to participate in this study was provided by the participants’ legal guardian/next of kin.

## Author Contributions

RW conceptualization and writing review and editing, project administration, and funding acquisition. HL supervision. HW investigation and writing original draft. ZW formal analysis and investigation. YY resources, review, editing, and validation. All authors contributed to the article and approved the submitted version.

## Conflict of Interest

The authors declare that the research was conducted in the absence of any commercial or financial relationships that could be construed as a potential conflict of interest.
